# Multiband Semimetallic Electronic Structure of Superconducting Ta_2_PdSe_5_

**DOI:** 10.1371/journal.pone.0123667

**Published:** 2015-04-24

**Authors:** David Joseph Singh

**Affiliations:** 1 Materials Science and Technology Division, Oak Ridge National Laboratory, Oak Ridge, Tennessee, USA; Boston College, UNITED STATES

## Abstract

We report the electronic structure and related properties of the superconductor Ta_2_PdSe_5_ as determined from density functional calculations. The Fermi surface has two disconnected sheets, both derived from bands of primarily chalcogenide *p* states. These are a corrugated hole cylinder and a heavier complex shaped electron sheet. The sheets contain 0.048 holes and a compensating number of electrons per formula unit, making the material a semimetallic superconductor. The results support the presence of two band superconductivity, although a discrepancy in the specific heat is noted. This discrepancy is discussed as a possible consequence of Pd deficiency in samples.

## Introduction

Superconductivity with very large upper critical fields has been reported in several layered compounds *M*_2_Pd_*x*_ *Ch*_5_, *M* = Nb, Ta and *Ch* = S, Se. [1–8] The upper critical field is reported to exceed an estimate of the Pauli limit [9, 10] for Ta_2_Pd_0.92_S_5_. [1] This has been discussed both in terms of spin-orbit effects, [1, 5, 6] and multiband superconductivity. [4, 11, 12]

An important chemical feature of these compounds is that one of the Pd sites in the unit cell is chemically less favorable than the other, leading to a tendency for Pd deficiency. [1, 11] It was argued that, although the average crystal structures are centrosymmetric, this Pd deficiency can lead to local inversion symmetry breaking, which with heavy elements such as Ta or Pd in the structure can lead to enhancement of the upper critical fields from spin-orbit scattering. [1, 5, 6] The fact that the samples invariably have large number of Pd vacancies is consistent with this view. In this regard, the metal atoms occur on one dimensional chains in the crystal structure, which has been emphasized in relation to spin-orbit effects. [6]

The large upper critical fields have also been discussed in terms of multiband superconductivity. In particular, it was noted that the electronic structure of Ta_2_PdS_5_ has two main bands, and in addition there is evidence for strong coupling, which together could also account for the high upper critical fields. [11] Multiband superconductivity has also been discussed in the related compounds Nb_3_Pd_0.7_Se_7_, [13] and Ta_4_Pd_3_Te_16_. [14] The band structures do show multiple Fermi surfaces consistent with this view and a recent report of the specific heat of Nb_2_Pd_*x*_S_5_ with different applied fields is consistent with two different superconducting gaps. [12]

One way forward in understanding the high upper critical fields and other properties of these materials is through the systematic property variation between different compounds and samples with different stoichiometry in relation to band structure calculations. Recently, Zhang and co-workers, [4] have reported a new system, Ta_2_PdSe_5_, which is superconducting with critical temperature *T*_*c*_ = 2.5 K, and has a low temperature dependence of the specific heat consistent with multiband superconductivity. This newly reported compound has an upper critical field *H*_*c*2_(0) = 15.5 T, for a ratio *H*_*c*2_(0)/*T*_*c*_ = 6.2, similar to the other compounds in the family. The purpose of the present paper is to elucidate the electronic structure of this compound in relation to experiment.

## Results

**Fig 1:**
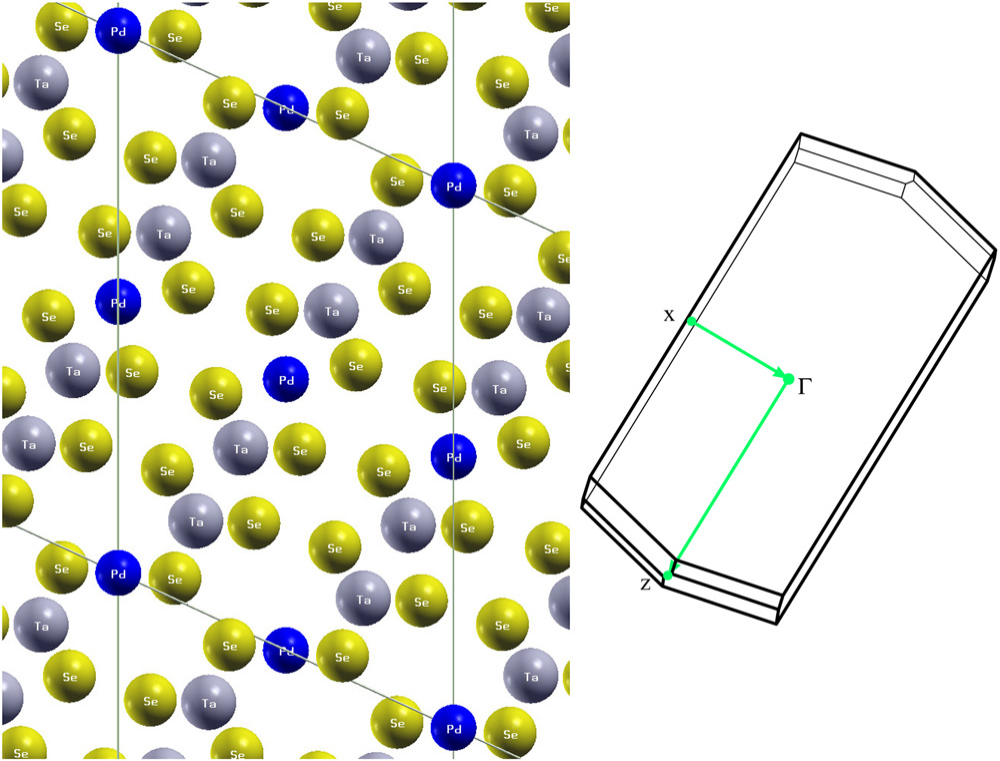
Crystal structure of Ta_2_PdSe_5_ with the relaxed atomic positions (left) and Brillouin zone (right). The crystal structure is viewed along the short *c*-axis, which is the direction of the metal chains. The conventional unit cell is indicated. The colored lines in the zone indicate the **k**-point path in the band structure plot.

The calculated atomic positions from total energy minimization at the experimental lattice parameters are given along with the bond valence sums in Table 1. The resulting structure is depicted in Fig 1. It follows the structure type as described by Squattrito and co-workers. [15] It is qualitatively similar to that of Ta_2_PdS_5_, described previously, [11] and in particular has a 4-fold coordinated Pd site connecting ribbons of composition Ta_4_PdSe_10_. This site is prone to vacancies in the sulfide. Deviation from the nominal stoichiometry has not been reported in Ta_2_PdSe_5_, but the possibility of Pd vacancies associated with the Pd2 site should be kept in mind. The shortest metal-metal distances are along the *c*-axis, forming the one dimensional metal chains that have been discussed previously. [6] The metal atoms in these chains are separated by *c* = 3.117 Å. However, even from the point of view of metal-metal bonding, the structure may not be best viewed as one-dimensional. Specifically, there are comparable in-plane metal-metal distances. For example, the Ta2-Pd2 distance is 3.229 Å. Importantly, the Pd2 site joins the ribbons, mentioned above, and depending on the electronic hopping, the ribbons might or might not be independent one dimensional objects from an electronic point of view. The bond valence sums deviate substantially from nominal ionic values, except for Ta. The average Se value is 2.95. These deviations are indicative of covalent bonding.

**Table 1:** Structure of Ta_2_PdSe_5_ based on relaxation of the atomic coordinates using the experimental lattice parameters, spacegroup 12, *C*2/*m*, *a* = 12.801 Å, *b* = 18.7554 Å, *c* = 3.117 Å, *γ* = 114.7266°. “b.v.” denotes the bond-valence sum.

atom	*x*	*y*	*z*	b.v.
Ta1	0.3648	0.3348	1/2	4.71
Ta2	0.2307	0.1618	0	5.04
Pd1	0.5000	0.5000	0	2.11
Pd2	0.0000	0.0000	0	1.84
Se1	0.3312	0.9618	0	2.90
Se2	0.4043	0.2070	1/2	3.18
Se3	0.5412	0.3827	0	2.87
Se4	0.7914	0.4145	1/2	2.73
Se5	0.6458	0.2319	0	3.09

**Fig 2:**
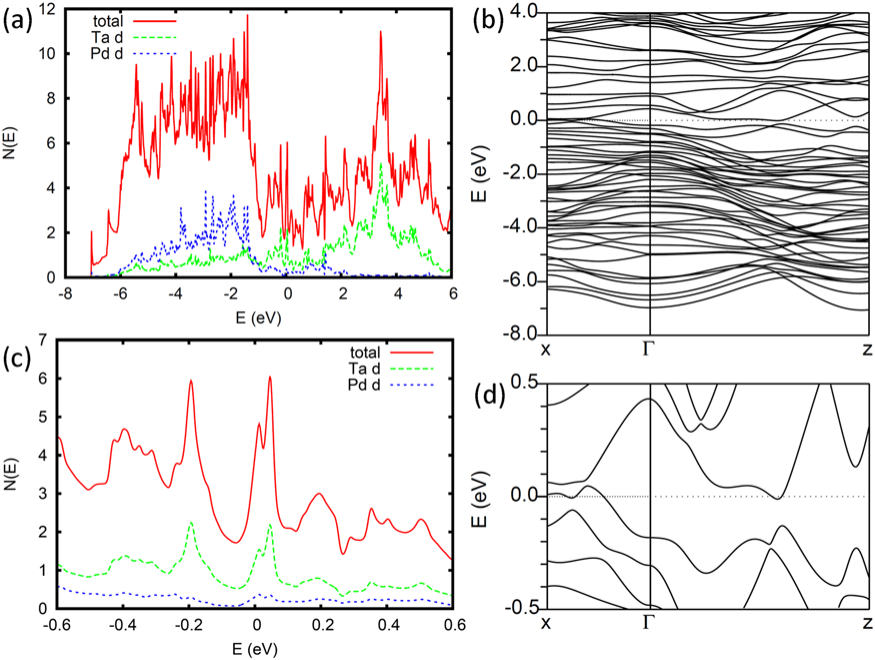
Electronic structure of Ta_2_PdSe_5_. (a) The density of states on a per formula unit basis. (b) The band structure along the directions given in Fig 1. (c) A blow-up of the density of states around *E*_*F*_, which is at 0 eV in all plots. (d) The band structure near *E*_*F*_.

The calculated electronic density of states and projections of Ta and Pd *d* character are shown in Fig 2, along with the band structure. As may be seen, the Ta *d* states occur mainly above the Fermi level, *E*_*F*_, while the Se *p* states and Pd *d* states are mainly below *E*_*F*_. The nearly fully occupied Pd *d* states argue against the presence of spin-fluctuations associated with Pd. In any case, a high degree of covalency is evident both from the Ta (Se) contributions below (above) *E*_*F*_ and from the width of the corresponding regions of the density of states.

The calculated density of states at the Fermi level is *N*(*E*_*F*_) = 3.94 eV^−1^ for both spins on a per formula unit basis, and as seen is mostly derived from Se *p* states. The metal *d* contributions are 0.86 eV^−1^, 0.38 eV^−1^, 0.23 eV^−1^ and 0.37 eV^−1^, for Ta1, Ta2, Pd1 and Pd2, respectively, on a per metal atom basis, for a total Ta *d* contribution of 1.24 eV^−1^ and a total Pd *d* contribution of 0.30 eV^−1^ per formula unit. These low numbers place the materials very far away from any transition metal magnetism. Furthermore, since superconductivity is an instability of the Fermi surface of a metal, it is clear that the electrons involved in pairing are in bands that are primarily derived from chalcogen *p* states, hybridized with mainly Ta1 *d* states.

The bare electronic specific heat coefficient from the calculated density of states is *γ*_*bare*_ = 9.6 mJ/(mol K^2^). This is very close to the measured *γ* = 10.3 mJ/(mol K^2^), which is not expected, since there should be an enhancement *γ* = (1+*λ*)*γ*_*bare*_. Here *λ* is roughly the superconducting electron-phonon coupling, *λ*_*ph*_ in the absence of spin-fluctuations, and even larger if there are substantial spin-fluctuation effects. The low value inferred from comparison of the calculated *γ*_*bare*_, with the experimental *γ* is not compatible with superconductivity. In the experimental data of Zhang and co-workers [4] the specific heat jump is found to be, Δ*C*_*e*_/*γT*_*c*_ ∼0.83, which is much smaller than the weak coupling value of 1.43. Zhang and co-workers note that this could be due to a non-superconducting fraction in the sample. A multiphase sample could also affect the inferred value of *γ*, for example if one of the phases has a small *γ*. Another likely explanation is that off-stoichiometry, e.g. Pd vacancies is important. *E*_*F*_ lies on the leading edge near the top of a peak in the density of states, which means that broadening due to disorder would be expected to lower *N*(*E*_*F*_). Metal deficiency, if it lowers *E*_*F*_ would also reduce *N*(*E*_*F*_).

**Fig 3:**
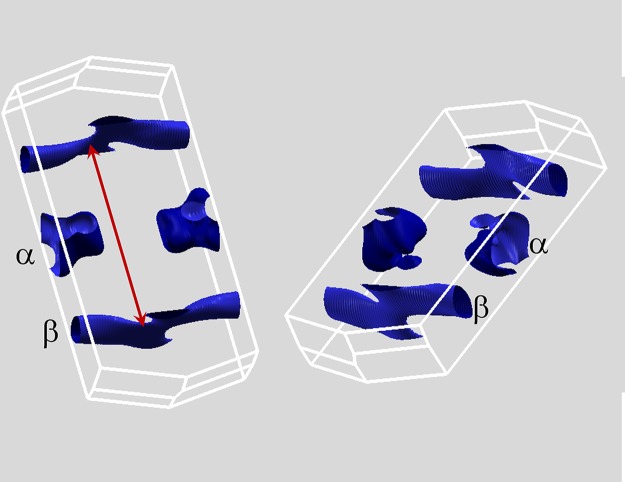
Calculated Fermi surface of stoichiometric Ta_2_PdSe_5_. Two views are shown. The zone is as depicted in Fig 1. The two sheets, *α* and *β* are as indicated. The red line denotes the nesting vector for the *β* sheet.

The Fermi surface is depicted in Fig 3. It has two disconnected sheets. Such a structure can be very favorable for superconductivity with sign changes of the order between the sheets if there are repulsive interactions, e.g. spin-fluctuations, that couple the sheets. [16–19] However, this is highly unlikely here because, as mentioned, the bands near *E*_*F*_ have relatively little transition metal character, placing the material far from magnetic instabilities, while the electron-phonon interaction is attractive. The Fermi surface consisting of two disconnected sheets also allows for multiband superconductivity, which was proposed as an explanation for the high *H*_*c*2_)(0) values in this family of materials, [11] following the general arguments of Gurevich and co-workers. [20–23] The two sheets of Fermi surface are a hole sheet, *α*, in the form of a very corrugated cylinder around the point labeled *x* (Fig 1) and a compensating complex shaped electron surface, *β*, consisting of interconnected tube shaped sections. These *β* sheets are flat along the direction Γ-*z*, and are therefore nested as shown by the line in Fig 3. The *α* and *β* Fermi surfaces each contain ∼0.048 of the zone. Therefore there are 0.048 holes per formula unit (two formula units per cell) in the *α* pocket and a corresponding number of electrons in the *β* pocket. Ta_2_PdSe_5_ is thus a semimetallic superconductor, as are the Fe-based superconductors. [24] The electron and hole Fermi surfaces contribute ∼40% and 60%, respectively, to *N*(*E*_*F*_), i.e. the electrons are heavier than the holes in this compound.

Neither of the two sheets of Fermi surface is one dimensional (a one dimensional surface associated with the metal chains would be a flat section perpendicular to the Γ-z direction as labeled in Fig 1). Both of the Fermi surfaces are associated with bands near an anticrossing. This leads to complex shaped bands very near *E*_*F*_, which may lead to a particularly strong sensitivity to disorder. This may be present due to Pd vacancies. The cylindrical hole sheet is associated with the inverted band at the corresponding face of the zone. The band shape is interesting from a topological point of view because of this band inversion and because the position on the zone face yields a one pocket per zone, i.e. an odd number of pockets. In fact, the complex shapes of the Fermi surfaces in spite of the low band fillings, are a direct consequence of the origin in band inversion with spin-orbit as discussed recently in the context of thermoelectric materials. [25]

The conductivity tensor scaled by the scattering rate was determined from the Fermiology within the constant scattering time approximation. This assumes that the scattering rates on the two sheets of Fermi surface are the same. The resulting eigenvalues of *σ*/*τ* were 4.4×10^18^ 1/(Ωms) and 8.7×10^18^ 1/(Ωms) in the *a*, *b*, plane, and 3.10×10^19^ 1/(Ωms) along the *c*-axis direction. Thus the conductivity is highest along the *c*-axis, which is the direction of the ribbons and metal atom chains. However, the anisotropy of ∼7 between the highest and lowest conductivity directions is not large enough to reasonably call this a one dimensional conductor.

## Discussion

We report calculations of the structure and electronic properties of the superconductor Ta_2_PdSe_5_. Perhaps not surprisingly, the calculations show this compound is in many respects very similar to previously reported members of this family such as Ta_2_PdS_5_. The calculated density of states is only slightly smaller than the value inferred from the experimental specific heat, which is not expected for a superconductor. This discrepancy, which might be due to Pd vacancies in the samples, suggests further study.

Importantly, Ta_2_PdSe_5_ is an anisotropic three dimensional metal with two disconnected compensating sheets of Fermi surface. This is consistent with the explanation that the high upper critical fields in these compounds are a manifestation of multiband superconductivity. The calculations do not support the view that this material is reasonably described as a one dimensional metal. Both the Fermi surface sheets occur from bands near anticrossings. This leads to a topological structure for the band forming the hole cylinder.

Multiband superconductivity is a much discussed subject, [20, 26–28] but has a limited number of established examples. These include unconventional superconductors, particularly the Fe-based superconductors, [23] and the electron-phonon superconductor MgB_2_. [29, 30] Ta_2_PdSe_5_ and the related chalcogenides provide a new family of materials exhibiting multiband electron-phonon superconductivity. The relatively accessible values of the upper critical fields and the variety of compounds in this family should make these compounds particularly useful for studying multiband superconductivity.

## Methods

The electronic structure calculations were performed within density functional theory using the generalized gradient approximation of Perdew, Burke and Ernzerhof (PBE). [31] The calculations were performed using the general potential linearized augmented planewave (LAPW) method, [32] as implemented in the WIEN2k code. [33] The standard LAPW linearization was employed with local orbitals [34] to treat the semicore states, specifically the 5*s* and 5*p* states of Ta, the 4*p* states of Pd and the 3*d* states of Se. LAPW sphere radii of 2.4 bohr, 2.3 bohr and 2.3 bohr were used for Ta, Pd and Se, respectively.

Well converged basis sets were employed. The planewave cutoff, *k*_*max*_ was determined by *R*_*min*_ *k*_*max*_ = 9, where *R*_*min*_ = 2.3 bohr was the smallest sphere radius. The crystal structure was based on the experimental monoclinic lattice parameters, in space group 12. [4] The standard *C*2/*m* setting was used for convenience, similar to prior calculations for the sulfide, Ta_2_PdS_5_. [11] The internal atomic coordinates were determined by total energy minimization keeping the lattice parameters fixed at the experimental values. This was done in a scalar relativistic approximation. The electronic structures and related properties were then calculated including spin-orbit. The results are based on dense Brillouin zone samplings of ∼5900 points in the irreducible wedge. The conductivity anisotropy was determined using the BoltzTraP code as applied to the electronic structure including spin-orbit. [35]
